# Production of cellulase from *Aspergillus terreus* MS105 on crude and commercially purified substrates

**DOI:** 10.1007/s13205-016-0420-z

**Published:** 2016-04-13

**Authors:** Muhammad Sohail, Aqeel Ahmad, Shakeel Ahmed Khan

**Affiliations:** Department of Microbiology, University of Karachi, Karachi, 75270 Pakistan

**Keywords:** *Aspergillus terreus*, Endoglucanase, β-glucosidase, Solid-state fermentation, Crude substrates

## Abstract

*Aspergillus terreus* MS105 was originally isolated from soil and screened for cellulase production in the presence of various carbon sources including carboxymethyl cellulose (CMC), avicel, sigmacell, filter-paper and salicin. CMC induced the production of endoglucanase (EG) and filter-paperase while the levels of β-glucosidase (BGL) were increased when salicin was present in the medium. Nature of production medium influenced the duration of lag- and log-phase of the growth, rate of fungal dry-mass and enzyme production. The volumetric and specific productivity of cellulase under submerged fermentation of grass were 1.7–20-folds higher than sugarcane-bagasse, corncob and commercially available purified substrates. Nonetheless, solid state fermentation (SSF) of crude substrates also yielded high volumetric productivity of EG and BGL. The studies on characterization of enzymes showed that EG was more thermostable than BGL with an optimum activity at 70 °C and a melting temperature of 76 °C. A 1.2–1.5-folds increase in EG activity was observed in the presence of K^+^, Ca^2+^ and Mg^2+^, whereas, the EG and BGL activities remained unaffected in the presence of EDTA. Both the enzyme activities performed optimally under acidic range of pH.

## Introduction

Cellulose is a linear polymer of glucose residues linked together by β-1-4 linkages. There can be several thousands of chains comprising microfibrils in plant cell-wall (Beguin and Aubert 1994). With an annual increment of millions of tons, the polymer is considered to be a renewable source of chemicals and energy; hence its degradation has remained a subject of intense research. In addition to complex arrangement of microfibrils through hydrogen bonding, the presence of lignin and hemicelluloses in plant cell-wall hinders the hydrolysis of cellulose. Physico-chemical hydrolysis of lignocellulosics (LC) has remained limited to pretreatment processes such as acidic, alkaline, steam explosion and ammonia fiber explosion (Wyman et al. 2005). The biological pretreatment employs microorganism or microbial enzymes (Kumar et al. 2016), however, the ability of fungal enzymes to degrade LC biomass efficiently has diverted the attention of researchers to enzymatic conversion of LC (Bhat 2000). Being the most abundant polymer present in LC substrates, cellulose is termed as a renewable feedstock of chemicals and energy and hence its biological degradation is a subject of intense research. The complete enzymatic degradation of cellulose requires the concerted action of three enzymes (Lynd et al. 2002), 1,4-D-glucan-4-glucanohydrolase or endoglucanase (EG), 1,4-D-glucan-glucohydrolase or exoglucanase (EX) and D-glucoside-glucohydrolase or β-glucosidase (BGL).

Genes encoding cellulases are dispersed throughout microbial taxonomic groups (Lynd et al. 2002), nonetheless, fungal species are employed industrially for cellulase production and for the conversion of LC (Lynd et al. 2005). The hyphal mode of growth coupled with the ability to produce a battery of hydrolytic enzymes make fungi a suitable choice for the degradation of LC. Fungal strains have long been applied for the degradation of plant based biomass and to produce valuable commodities using crude substrates from plants. Additionally, the fungal enzymes produced on crude LC substrates find an array of biotechnological applications from diagnostics to food and biofuel production (Bhat 2000). Indeed, fungal oxidative and hydrolytic enzymes are being applied for environmental management and biomass degradation (Kues 2015).

Despite several commercial and industrial applications, the enzyme market always finds a competitive disadvantage due to higher production cost of enzymes. There are different strategies which are being sought to reduce the cost of enzyme production; use of agricultural and domestic residues in place of costly fermentation media is one of the widely adopted strategy.

Agricultural waste and residues including sugarcane-bagasse (Siqueira et al. 2013), grasses and corncob (Pointner et al. 2014) have been recognized as feasible substrates particularly for the production of fungal enzymes. Huge amounts of these substrates remain available throughout the year (Wyman et al. 2005) in most of the agricultural countries, including Pakistan (Arshad and Ahmed 2016) and hence can be utilized to develop fermentation industries. The higher contents of cellulose (32–43 % in grasses, 35–45 % in sugarcane-bagasse and 38–69 % in corncob) render them promising substrates for the production of cellulases (Pointner et al. 2014; Siqueira et al. 2013; Ververis et al. 2004).

Although cellulase preparations from *Trichoderma* species are commercially available, they lack the significant levels of BGL and hence the enzyme system faces feedback inhibition. The enzyme cocktails from *Aspergillus* species are used alternatively (Gusakov 2011), though with a lower titers but contains higher activities of BGL. Plants cell wall degrading enzymes (CWDE) from members of this genus have been extensively studied and genomic organization of many of the enzymes has been reported (De Vries and Visser 2001) that led to the development of many recombinant strains. Industrially, *A. niger* is often used for cellulase production while, *A. terreus* is used in the production of cholesterol lowering drug (Kumar et al. 2014). The production of cellulases from *A. terreus* has also been reported, but only few studies describing the production of cellulolytic enzymes under SSF of crude LC substrates have appeared so far. Earlier, the production of various enzymes from *A. terreus* MS105 on banana peels was reported (Rehman et al. 2014). However, in the present work the production of cellulases on grass, sugarcane-bagasse and corn-cob is described.

## Materials and methods

### Strain and cultivation medium

The strain *Aspergillus terreus* MS105, previously reported for the production of cellulases (Sohail et al. 2009a) was retrieved from the culture collection of the Department of Microbiology, University of Karachi. It was maintained and sub-cultured on Sabauraud’s dextrose agar (SDA; Oxoid).

### Solid state fermentation

The inoculum was prepared by growing the strain MS105 at 35 °C on SDA plates for five days. Spore suspension containing 5 × 10^7^ spores/ml was prepared in sterile saline.

Natural substrates, grass, corncob, sugarcane-bagasse were obtained, washed, dried and ground to 100 mesh size and stored at room temperature in sealed glass bottles, until used. SSF was carried out by autoclaving 2 g of the substrate at 121 °C for 30 min and moistened to 65 % with mineral salt medium (MSM; Mandels and Weber 1969). It was inoculated with 2 ml of spore suspension and incubated at 35 °C for a suitable period. To harvest crude enzyme preparation, 50 ml of the sodium citrate buffer (50 mM, pH 4.8) was added to fermented substrate and agitated to 150 rpm for 2 h. The slurry was filtered through four layers of muslin cloth and Whatman # 1 filter-paper, followed by centrifugation at 5000*g* for 20 min. The resultant cell-free culture supernatant (CFCS) was used as crude enzyme preparation.

### Submerged fermentation

Initially, enzyme production was studied in mineral salt medium (MSM) containing commercially available cellulosic substrates including carboxymethyl cellulose (CMC), filter paper (FP), cellulose acetate (CA), sigmacell (SC), salicin, ball-milled cellulose (BMC) and phosphoric-acid swollen cellulose (PASC) and compared with the production of enzymes in the presence of glucose in MSM and in Sabauraud’s dextrose broth (SDB).

The growth and enzyme production kinetics was carried out in 250 ml Erlenmeyer flask containing 100 ml of MSM supplemented either with 1 % crude substrate or 0.5 % commercially available substrates. The spore suspension (*A. terreus* MS105) was inoculated to the medium (pH 5.5), incubated in a shaking incubator at 35 °C, 200 rpm for a suitable time period. It was followed by the separation of fungal mass from MSM containing soluble substrate by filtration, as mentioned earlier for SSF and dry-mass determined to study the growth kinetics. The growth kinetics data on commercially available soluble substrates was compared with the data obtained from SDB. The CFCS was used to investigate enzyme production kinetics. Growth was not monitored in the presence of insoluble natural substrates.

### Enzyme assays and enzyme characterization

Filter-paperase, EG and BGL activities were determined by incubating 1 ml of CFCS with 1 ml of 50 mM sodium citrate buffer (pH 4.8) containing filter-paper strips (6 × 1 cm), 0.5 % CMC or 0.5 % salicin, respectively, for 30 min at 50 °C followed by determination of reducing-sugars (Miller 1959). One unit of the enzyme activity was defined as the amount of enzyme that liberates one micromole of reducing sugar (as glucose equivalent) in one minute from the substrate under standard assay conditions. The optimum temperature for the enzyme activity was determined by performing enzyme assays at different temperatures (30–80 °C). Likewise, optimum pH for enzyme activity was determined using different buffers for enzyme assays, i.e., 50 mM HCl-KCl buffer (for pH 1–2), 50 mM Glycine–HCl buffer (for pH 2.5–3.5), 50 mM sodium acetate buffer (for pH 4.0–5.5), 50 mM citrate phosphate buffer (for pH 6.0–7.0), 50 mM TRIS–HCl buffer (for pH 7.5–9.0) and 50 mM Glycine-NaOH buffer (for pH 9.5–10.0). To determine melting-temperature (*T*_m_) for enzymes, CFCS was incubated at temperatures ranged between 50 and 80 °C for 15 min followed by assaying the residual enzyme activity. The activity of untreated CFCS was considered as 100 % and used to calculate relative activity of treated CFCS. The effects of metallic ions and EDTA were studied by performing enzyme assays with dialyzed CFCS in the presence of 20 and 50 mM of mineral salts or EDTA.

## Results

The cellulolytic potential of *A. terreus* MS105 was studied by growing fungus in mineral salt broth containing various substrates. The data showed that the strain produced EG and BGL on tested cellulosic substrates (Table 1). The strain yielded the highest titers of filter-paperase and EG in CMC containing media while the highest level of BGL was observed in the presence of salicin. The fungus was unable to produce any detectable amount of cellulolytic enzymes in glucose containing media.

**Table 1:** Production of filter-paperase (IFPU), endoglucanase (EG) and β-glucosidase (BGL) by *A. terreus* MS105 in mineral salt medium (MSM) containing either 1 % (w/v) carboxymethyl cellulose (CMC), ball-milled cellulose (BMC), phosphoric acid swollen cellulose (PASC), filter paper (FP), cellulose acetate (CA), sigmacell (SC), or salicin and 2 % (w/v) glucose in sabauraud’s dextrose broth (SDB)

Media	Enzyme titers (IU/ml)		
	IFPU	EG	BGL
MSM + CMC	0.295	0.47	0.30
MSM + BMC	0.184	0.32	0.15
MSM + PASC	0.08	0.12	0.07
MSM + FP	0.15	0.28	0.18
MSM + CA	0.10	0.18	0.08
MSM + SC	0.11	0.26	0.16
MSM + Salicin	0.22	0.38	0.56
MSM + Glucose	0	0	0
SDB	0	0	0

**Fig. 1:**
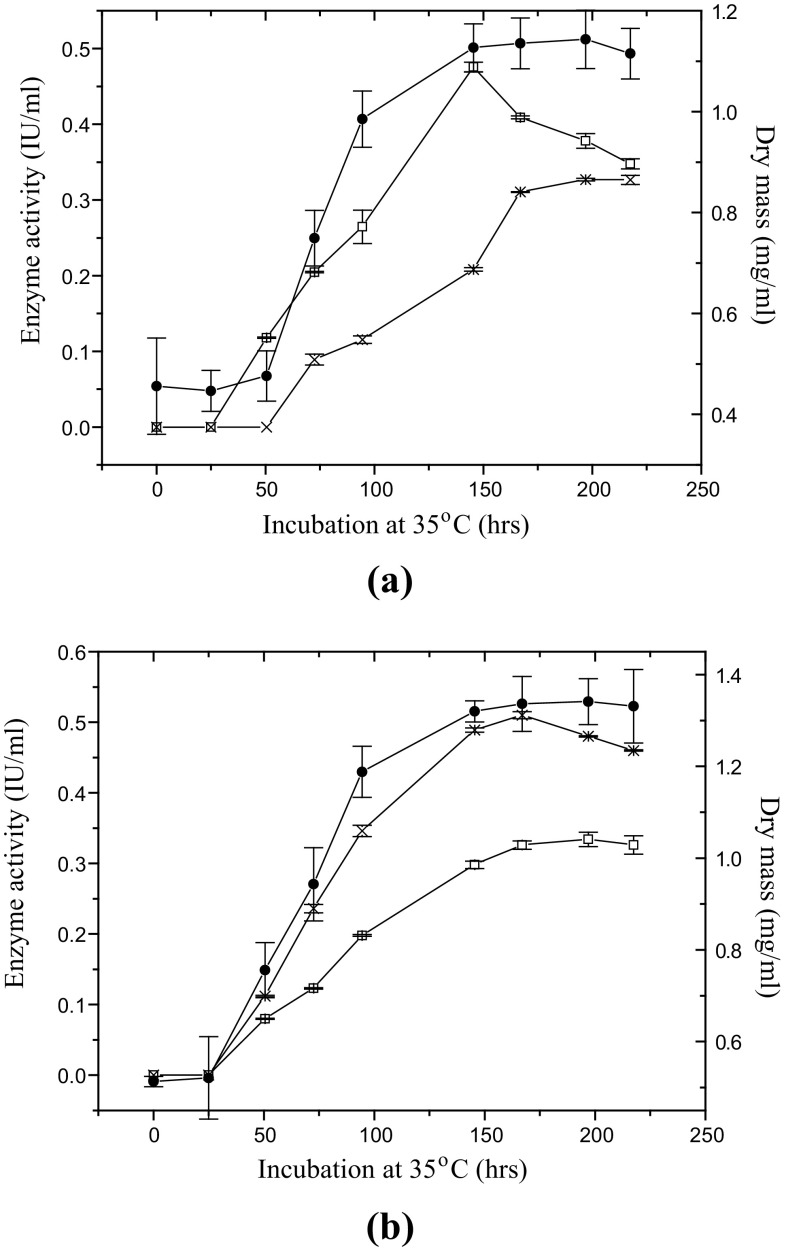
Dry mass (*x*), Endoglucanase (*square*) and β-glucosidase (*filled circle*) production kinetics in mineral salt medium (MSM) containing, **a** 1 % (w/v) CMC and **b** 1 % (w/v) Salicin

Since, the enzyme production by MS105 was favored in the presence of CMC and salicin, therefore, growth and enzyme production kinetics were investigated in MSM supplemented with these substrates and compared with MSM containing glucose or SDB. The results revealed that the presence of glucose in MSM or in SDB forced the fungus to rapidly enter in long log-phase (data not shown), consequently, the generation time was decreased to 1.4–2.6 h (Table 2); it led to the formation of more dry cell-mass and yielded higher biomass productivity. Whereas, CMC (Fig. 1a) and salicin (Fig. 1b) being complex sources of carbon, causing a delay in the commencement of log-phase, the generation time was increased. A shorter lag-phase and higher cell-density differentiated the growth in salicin containing medium compared to the growth in CMC supplemented medium. However, the production of EG and BGL in the presence of both the inducers, i.e. CMC or salicin was initiated with the onset of the log-phase of the growth and attained its peak in stationary-phase. Moreover, the volumetric and specific productivity of EG was 1.6–1.7-folds higher in CMC containing medium than in salicin supplemented medium (Table 3) while, BGL productivity was enhanced in the presence of salicin.

**Table 2:** Summary of growth-kinetics indicating the duration of lag- and log-phase, volumetric biomass production (*Q*_x_) and generation time (*g*) when *A. terreus* MS105 was grown in Mineral salt medium (MSM) containing 1 % (w/v) carboxymethyl cellulose (CMC) or salicin or 2 % (w/v) glucose and in sabauraud’s dextrose broth (SDB)

Substrate/medium	Duration (h)		g (h)	*Q*_*x*_ (mg/h)
	Lag phase	Log phase		
MSM + CMC	50	90	14.601	0.068
MSM + Salicin	20	110	15.081	0.066
MSM + Glucose	5	50	2.651	0.385
SDB	5	40	1.478	0.672

**Table 3:** Volumetric and specific productivity of endoglucanase (EG) and β-glucosidase (BGL) from *A. terreus* MS105 cultivated in 1 % (w/v) CMC or salicin supplemented MSM

Substrate	Product formation parameters			
	Volumetric productivity (IU/l/h)		Specific productivity (IU/mg of cells)	
	EG	BGL	EG	BGL
CMC	0.327	0.185	0.709	0.457
Salicin	0.195	0.305	0.396	0.619

One of the major aims of the present study was to explore the potential of *A. terreus* MS105 to utilize crude lignocellulosic (LC) substrates for the production of cellulolytic enzymes. The strain was cultivated under submerged conditions in MSM supplemented with grass, corn-cob and bagasse (as sole C-source) and enzyme productivity was compared with the growth of strain MS105 on commercially purified substrates. The data revealed that the strain MS105 commenced the production of EG and BGL under submerged fermentation (Smf) of grass after 25 h that was much earlier than the duration taken by MS105 to initiate the enzyme production under Smf of other crude and commercially available substrates (data not shown). Furthermore, the enzyme titers obtained on grass were higher than the titers obtained on all other commercially available celluloses (Table 4). Also, the volumetric and specific productivity on grass was 1.7–20-folds higher than the productivity obtained from other substrates. The potential of the fungal strain MS105 to utilize untreated crude substrates can be explored for cost-effective production of cellulases on an industrial scale.

**Table 4:** Volumetric-(*Q*_p_) and specific-(*Y*_p/s_) productivity of endoglucanase (EG) and β-glucosidase (BGL) from *A. terreus* MS105 under Smf of crude and purified cellulosic substrates in mineral salt medium (MSM)

Substrate	*Q*_p_ (IU/l/h)		*Y*_p/x_ (IU/g substrate)	
	EG	BGL	EG	BGL
Grass	3.3604	8.301	7.123	17.6
Corncob	0.863	0.297	1.83	0.63
Sugarcane-bagasse	0.726	0.1603	1.543	0.34
Sigma cell	2.15	1.446	4.56	3.06
Cellulose acetate	0.948	0.970	2.01	2.05
Filter paper	0.9886	1.163	2.096	2.46
Avicel	2.037	1.305	4.32	2.766
Phosphoric-acid swollen cellulose	2.15	5.5	4.55	8.95

On the other hand, when MS105 was grown in the presence of sugarcane-bagasse and corn-cob, EG and BGL production was not detected until 50 h (data not shown). A similar pattern was also observed when Smf of commercially available substrates was studied except for phosphoric-acid swollen cellulose (PASC) and filter paper.

**Fig. 2:**
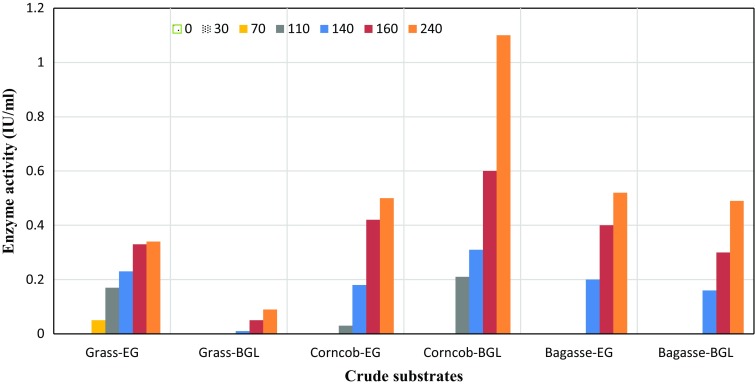
Comparative production of endoglucanase (EG) and β-glucosidase (BGL) under SSF of grass, corncob and sugarcane-bagasse

Interestingly, a different pattern of utilization of crude substrates was noted when these substrates were fermented in the absence of free-water, i.e. under SSF (Table 5). Under this condition, the fermentation of sugarcane-bagasse and corn-cob yielded more titers of EG and BGL, respectively, than on grass. The higher specific productivity of both the enzymes indicates the ability of the strain MS105 to utilize the crude substrate effectively; though, it took longer time to produce the enzymes and hence lowered rates of enzyme production were noted. The production kinetics data indicate that EG and BGL were not detected until 110 h of fermentation and attained their peak after 240 h (Fig. 2). Further incubation resulted a decline in enzyme titers (data not shown); that may be attributed to denaturation of the enzymes or to the activity of proteases produced by MS105. Nonetheless, the levels of EG and BGL produced under SSF were much lower than under Smf.

**Table 5:** Rate of endoglucanase (EG) and β-glucosidase (BGL) production and specific productivity (*Y*_p/x_) by *A. terreus* MS105 under SSF of crude substrates

Substrate	Rate of enzyme production (IU/h)		*Y*_p/x_ (IU/g substrate)	
	EG	BGL	EG	BGL
Grass	0.07	0.018	8.5	2.275
Corn-cob	0.104	0.241	12.591	29.0
Sugarcane-bagasse	0.111	0.108	13.83	12.96

EG from *A. terreus* MS105 retained 80 % of its activity (data not shown) when enzyme reaction was carried out between 50 and 60 °C and performed its activity optimally at 70 °C (Table 6). On the other hand, 90 % of BGL activity was noted at temperatures between 40 and 50 °C with an activity optima at 60 °C. Similarly, the *T*_m_ for EG (76 °C) was found to be much higher than BGL (60 °C). In addition, the optimum pH for both the enzymes falls between pH 4.0 and 6.0.

**Table 6:** Characterization of *A. terreus* MS105 endoglucanase (EG) and β-glucosidase (BGL) activities

	EG	BGL
Optimum temperature	70 °C	60 °C
Optimum pH	6.0	4.0
Melting temperature (*T*_m_)	76	60
Activators	K^+^, Ca^2+^, Mg^2+^	–
Inhibitors	Cu^2+^, Co^2+^, Hg^2+^, Ba^2+^, Fe^3+^, Ag^+^	Cu^2+^, Co^2+^, Hg^2+^, Ba^2+^, Fe^3+^, Ag^+^, Ni^2+^

EG and BGL activities were also assayed in the presence of various mineral salts and EDTA. The presence of Ca^2+^ or Mg^2+^ enhanced the overall activity of EG by 15–20 % (Table 6), whereas, none of the metallic ions found to be activator of BGL. The activity of both the enzymes were not altered in the presence of EDTA, indicating that divalent ions are not essentially required for their activities.

## Discussion

The potential of the strain MS105 to produce cellulase was initially evaluated in the presence of commercially available soluble substrates. It was noted that the production of EG and BGL was inducible as the enzymes were not detected when the strain MS105 was cultivated in glucose containing media. Induction of EG and BGL has already been reported for *Alternaria* sp. (Sohail et al. 2011) and for *A. niger* MS82 (Sohail et al. 2009b). It was noted that the rates of biomass and enzyme production were influenced greatly by the specific substrate in the medium. For instance, the rate of biomass productivity was lowered together with an increase in EG titers in the presence of CMC; it confirms the role as inducer of this substrate. Similar phenomenon was also observed for the production of BGL in the presence of salicin. Consequently, the higher (volumetric and specific) productivity of both the enzymes from the strain *A. terreus* MS105 reveals the inducibility of these enzymes. Conversely, a strain of *Trichoderma viride* reportedly produced 0.5 IU/ml of CMCase activity in the presence of glucose, sucrose, xylose and CMC, however, CMC was proved to be the most potent inducer of FPase activity (Nathan et al. 2014).

The use of purified substrates for the production of enzymes is uneconomical and hence the cost of enzyme production can be reduced using crude substrates (Lakshmi et al. 2009) particularly under SSF. Though the SSF is an old technology, the utilization of waste materials with generation of lesser effluents renewed researchers’ interests in this process. The selection of substrate that can facilitate anchorage of fungi, along with providing sufficient nutrients under SSF processes remained a subject of intense research for many decades (Yoon et al. 2014). Previously, *A. terreus* MS105 has been studied for its potential to produce cellulases and other plant CWDE under SSF of banana-peels in mono-and in co-culture with *A. niger* (Rehman et al. 2014); in this study cellulolytic potential of *A. terreus* MS105 was evaluated under Smf as well as in SSF of grass, corn-cob and sugarcane-bagasse. The strain produced the highest titers of EG and BGL under Smf of grass and SSF of sugarcane-bagasse indicating the potential of this strain to utilize a variety of substrates in the presence and in the absence of free-water. In one of the previous studies, Gao et al. (2008) described corn-stover as one of the most suitable substrate out of all the other tested substrates for the production of cellulases from a thermophilic strain of *A. terreus* M11; however, SSF of sugarcane-bagasse yielded low titers of the enzyme by the strain M11. Naseeb et al. (2015) reported about the production of cellulase-free xylanase by *A. fumigatus* under SSF of sugarcane-bagasse and corn-leaves. The variable ratio of cellulose and hemicellulose in crude substrates regulates the production of cellulase and/or xylanase by the fermenting strains (Ghanem et al. 2000). Furthermore, the amount of other nutrients available and physiological potential of the strain also influence the production of enzyme under SSF as reported by Lakshmi et al. (2009) for xylanase.

The production of cellulases under SSF generally exhibits a lapse of 2–4 days at initial stage of the growth when the fungus colonizes the substrate and ensues its hydrolytic activity. As a result substrate depolymerizes and induces further production of enzymes (Yoon et al. 2014). A similar phenomenon was observed when it took longer time to produce enzymes by *A. terreus* MS105 under SSF than under Smf. This may also be endorsed to the production of other plant CWDE by the organism, as these can modify the LC substrate to an extent that cellulose become available to the fermenting organism. A requirement of 3 days of cultivation for the production of cellulase under SSF of corn-stover and wheat-bran by *A. niger, Trichoderma reesei* and *Penicillium oxalicum* was reported by Gong et al. (2015) indicating that many fungal species take longer periods to produce cellulases under SSF of crude substrates. However, Gao et al. (2008) mentioned 96 h as the optimum duration for the cellulase production from a thermoacidophilic strain of *A. terreus* M11; the modified substrate at elevated temperature and acidic pH might have caused an early commencement of enzymes production by the strain M11.

The application of crude enzyme preparations for the hydrolysis of agro-industrial wastes necessitates the investigation of effects of chemicals on enzyme activity. For instance, metallic ions can influence the enzymatic activities either by forming complexes with the substrate or by binding to side-chains of amino acids present in the active site (Blair, 1968). The data suggests the role of Co^2+^, Fe^3+^ and Ag^+^ as potent inhibitor of EG and BGL from *A. terreus* MS105. Whereas, both the enzymes remained unaffected in the presence of EDTA, showing that the enzymes do not require any divalent cation as cofactor. However, the activity of EG was enhanced in the presence of K^+^, Ca^2+^ and Mg^2+^ which was in line with an earlier finding for cellulases from *Alternaria* sp. (Sohail et al. 2011).

The application of cellulases in textile, saccharification of biomass and generation of cellulosic ethanol requires heat-stable enzymes. Heat stability can be studied by determining optimum temperature for activity or half-life at higher temperatures or by finding melting temperatures. Optimum temperatures within a range of 50-65 °C have been reported for EG from *Aspergillus niger* (Sohail et al. 2014), *Alternaria* sp. (Sohail et al. 2011) and *Cellulomonas biozotea* (Rajoka et al. 2003). Gao et al. (2008) reported about a decrease in EG and BGL activity from *A. terreus* M11 at pH above 5.0. The optimum temperature of 70 °C and *T*_m_ of 76 °C for EG from *A. terreus* MS105 under acidic pH suggests the possible exploration of this enzyme for high temperature biotechnological processes and in food-industries.

## Conclusions

*A. terreus* MS105 offers a wide range of advantages for its use in the production of cellulases. The strain is well adapted to ferment LC biomass in SSF and in Smf. The potential of the strain to produce higher titers of cellulases on crude substrates such as grass makes it a good candidate for an alternative to the strains that are currently employed in industries. Additionally, the higher optimum temperature for the activity of cellulase from this strain may provide a competitive edge over the other strains.
